# Cross-modal recognition of familiar conspecifics in goats

**DOI:** 10.1098/rsos.160346

**Published:** 2017-02-15

**Authors:** Benjamin J. Pitcher, Elodie F. Briefer, Luigi Baciadonna, Alan G. McElligott

**Affiliations:** 1 Biological and Experimental Psychology, School of Biological and Chemical Sciences, Queen Mary University of London, Mile End Road, London E1 4NS, UK; 2 Department of Biological Sciences, Faculty of Science and Engineering, Macquarie University, Sydney 2109 New South Wales, Australia; 3 Institute of Agricultural Sciences, ETH Zürich, Universitätstrasse 2, 8092 Zurich, Switzerland

**Keywords:** individual recognition, mammals, multimodal communication, ungulates, visual recognition, vocal communication

## Abstract

When identifying other individuals, animals may match current cues with stored information about that individual from the same sensory modality. Animals may also be able to combine current information with previously acquired information from other sensory modalities, indicating that they possess complex cognitive templates of individuals that are independent of modality. We investigated whether goats (*Capra hircus*) possess cross-modal representations (auditory–visual) of conspecifics. We presented subjects with recorded conspecific calls broadcast equidistant between two individuals, one of which was the caller. We found that, when presented with a stablemate and another herd member, goats looked towards the caller sooner and for longer than the non-caller, regardless of caller identity. By contrast, when choosing between two herd members, other than their stablemate, goats did not show a preference to look towards the caller. Goats show cross-modal recognition of close social partners, but not of less familiar herd members. Goats may employ inferential reasoning when identifying conspecifics, potentially facilitating individual identification based on incomplete information. Understanding the prevalence of cross-modal recognition and the degree to which different sensory modalities are integrated provides insight into how animals learn about other individuals, and the evolution of animal communication.

## 1. Background

Despite once being regarded as uniquely human [1], cross-modal recognition of individuals among non-human animals has received recent interest, with the aim of understanding how animals integrate information from multiple sensory modalities. Many species are capable of identifying conspecific as well as heterospecific individuals through single sensory modalities (e.g. [2–8]). However, the cognitive mechanisms underlying recognition are poorly understood [9]. The ability to integrate identity cues across sensory modalities would demonstrate the presence of higher-order cognitive representations that are independent of modality [9,10]. This may suggest that individuals form multimodal internal representations or templates of other individuals [10].

Cross-modal recognition has recently been examined in a small number of species and shown to include both auditory–visual and auditory–olfactory recognition of individuals. Auditory (e.g. vocal) and visual information are likely to be frequently encountered together because receivers see a calling individual when looking towards the source of a sound. Horses (*Equus caballus*) [9], crows (*Corvus macrorhynchos*) [11], African lions (*Panthera leo*) [12] and rhesus macaques (*Macaca mulatta*) [13,14] are capable of forming auditory–visual representations of conspecific individuals. This ability extends to heterospecific individuals in horses [15,16], rhesus macaques [14] and dogs (*Canis familiaris*) [17], which have all been shown to recognize familiar humans through audio-visual matching. In scent-marking species, auditory and olfactory information about an individual may be separated in space and time. Despite this, auditory–olfactory representations of conspecifics have been identified in lemurs (*Lemur catta*) [18]. Animals may also form cognitive representations of other individuals using multiple components of information from a single sensory modality. For example, golden hamsters (*Mesocricetus auratus*) integrate different scents from a given conspecific into a cohesive representation [19]. These studies suggest that some species are capable of integrating information across sensory modalities, including modalities that are not temporally or spatially linked. However, the extent to which familiarity between conspecific individuals influences cross-modal recognition has not been explored. Previous research has examined cross-modal recognition of familiar versus unfamiliar conspecifics (e.g. [11]), but have not addressed more subtle degrees of familiarity, such as close social affiliates versus ‘acquaintances’. If recognition, and particularly cross-modal recognition, is costly, animals may invest more in recognizing close social partners that are likely to be encountered more frequently, or over long periods of time, than those that are encountered infrequently.

Goats (*Capra hircus*) possess a number of characteristics that suggest they would benefit from advanced recognition abilities. Goats display good physical cognition abilities and long-term memory [20,21]. They also show basic social cognition, following conspecific gaze and human pointing to find hidden food [22], show audience-dependent human-directed behaviour in problem solving tasks [23] and learn socially from humans [24]. Further, goats are capable of some visual perspective taking, preferring to eat food that is out of the view of aggressive dominant individuals [25]. In the wild, goats live in complex, fission–fusion social groups where they forage in smaller groups during the day and aggregate in larger ‘night camps’ overnight, and these social groups have strong hierarchies [26–29]. Together, these traits indicate potential higher-order cognitive abilities in goats and the presence of complex social relationships that may require the ability to recognize a number of individuals.

Goat vocalizations provide listeners with a range of information about callers, including their physical characteristics, social group membership, individual identity and emotions [2,30–32]. Individual stereotypy of calls allows for individual vocal recognition, with both mothers and offspring displaying the ability to recognize each other using vocalizations alone [2]. Further, goats have long-term memory of individuals' vocalizations and vocal recognition may play an important role in social relationships, such as kin recognition and inbreeding avoidance [33]. Because of their important role in social interactions, it is likely that vocalizations form part of a cross-modal recognition system in goats.

The evidence for individual recognition in goats using visual cues is less clear than for auditory cues. Kids may use pelage pigmentation as a cue when searching for mothers in a herd, and are more prone to errors when presented with females with similar pelage coloration as their mothers [34,35]. Further, in social contexts, adult goats appear to use visual cues originating from an individual's body to discriminate social group from non-social group members [36]. While further controlled experiments of non-auditory recognition in goats are necessary, these studies imply that both visual and auditory cues are involved in individual recognition in goats and suggest the potential for cross-modal individual recognition.

In this study, we examined whether adult goats possess cross-modal representations (auditory combined with visual) of adult individuals that varied in their level of familiarity. Using a cross-modal preferential looking paradigm (e.g. [37,38]), we first presented goats with calls of either their stablemate (individual sharing their pen at night) or another familiar herd member in an arena where they could simultaneously observe both individuals. Secondly, we presented them with calls of one of two herd members, in order to test whether goats possess cross-modal representations of other, less familiar individuals than their stablemate. If goats were capable of integrating information across modalities, we predicted that upon hearing the call of an individual, they would look towards the congruent individual (the individual producing the call) faster and for longer than they would look towards the incongruent individual. Further, we predicted that familiarity would influence cross-modal recognition. While all animals in the study population were familiar to each other, we hypothesized that cross-modal recognition would be more developed among close social partners.

## 2. Material and methods

### 2.1. Experimental location and study animals

This study was conducted at Buttercups Sanctuary for Goats, Kent, UK (http://www.buttercups.org.uk), during June 2012. At the time, the sanctuary housed 125 domestic goats in a mixed herd of sexes and breeds (males in the herd are castrated, females are intact). At night, goats are housed in stables individually or in groups of two or three (average pen size = 3.5 m^2^) with straw bedding, within a larger stable complex. During the day, all goats are released together and can freely move between the stable complex and a large field (2 ha) containing several hay racks. Routine care of the animals is provided by sanctuary employees and volunteers. Goats have ad libitum access to hay, grass (during the day) and water, and are also fed with a commercial concentrate in quantities according to their state and age.

Ten goats (five females and five castrated males) were chosen as experimental subjects. These goats were fully habituated to human presence and had been used in previous studies [20,32,39,40]. For each subject we identified four individuals to be used as visual and audio stimuli during playbacks. These consisted of a ‘stablemate’ (sharing their pen at night) and three, non-stablemate, familiar individuals (herd member). Stablemates were social partners that were housed together at night in pairs with the subjects, seen closely associating during the day and never seen involved in agonistic interactions (E. Briefer 2012, personal observation; C. Nawroth 2012, personal communication). Herd members were also familiar individuals, but not housed with the subjects and not observed to be close social partners. Herd members were randomly chosen from the population. All the individuals had been housed at the sanctuary for at least 3 years prior to the experiment, allowing them to become familiar with other individuals. The pairs of stimulus goats used in each presentation were the same sex.

### 2.2. Auditory stimulus preparation

The contact calls of both the stablemates and herd members were recorded using a Sennheiser MKH 70 directional microphone in a Rycote Windshield and Windjammer, connected to a Marantz PMD 661 digital recorder (sampling rate of 44.1 kHz and amplitude resolution of 16 bits in WAV format). Calls were recorded in May 2012, approximately one month before the playbacks. Contact calls were recorded during 5 min of isolation in a familiar pen at the study site, as part of another study [41]. Recordings with good signal-to-noise ratios were used to construct playback presentations. Presentations were prepared using Adobe Audition 3 (Adobe Systems Incorporated, San Jose, CA, USA). All calls were normalized to 90% and saved as 44.1 kHz, 16 bit.wav format sound files for playback. Presentations consisted of two different contact calls (approx. 1 s duration each). Each call was followed by 10 s of silence (total duration, approx. 22 s). Playback presentations were made using an Edirol R-09 audio player at an approximately natural amplitude (76.7 ± 0.8 dB (mean ± s.e.)).

### 2.3. Experimental design and presentation arena

To examine cross-modal recognition, we used a preferential looking paradigm that is commonly used to examine cross-modal associations in humans and non-human animals [37,38]. This experimental design is based on the assumption that if an association exists between two cues, the presence of one cue will stimulate attention towards the other cue. Consequently, in the choice-test used in the present study, if cross-modal recognition existed, the presence of a vocal signal from a known individual was expected to trigger increased attention towards that individual in preference to the other individual.

**Figure 1.**
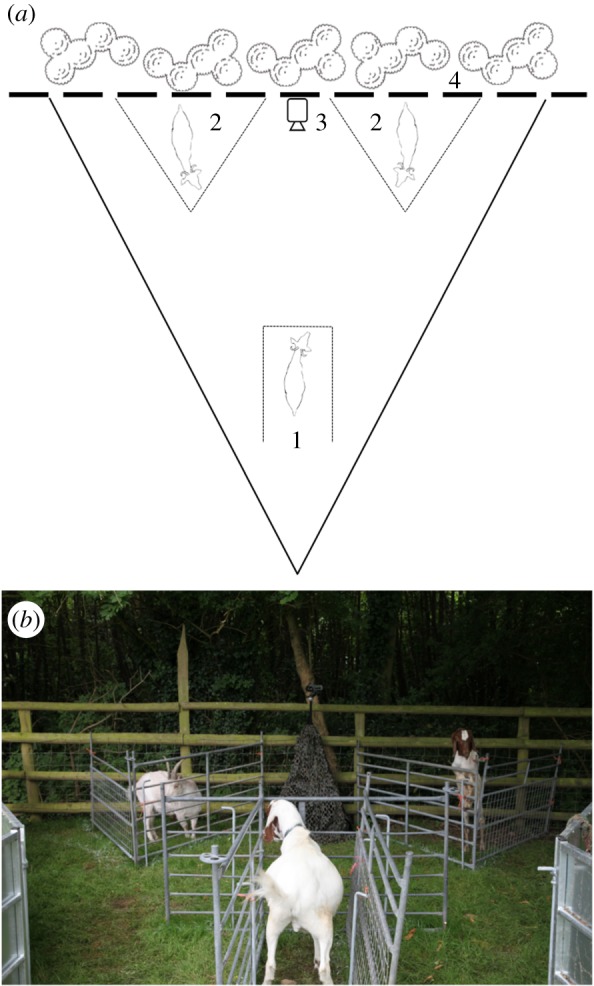
(*a*) Presentation arena schematic. The presentation arena was separated from the field by a solid metal fence (solid line). Within the arena, enclosures consisted of portable metal fencing with bars approximately 10 cm apart (dotted lines). The subject (1) was placed in the central enclosure after two stimulus goats (2) had been placed in the triangular enclosures. A camera and speaker (3) were located equidistant between the stimulus goats, facing the subject. The arena was located against a timber fence (dashed line) with vegetation behind (4) to prevent other animals from moving behind the stimulus and minimize visual distractions to the subject. (*b*) A photo of the presentation arena.

Playbacks were conducted in a triangular arena built within the large field surrounding the stable complex, where goats were released during the day (figure 1). The arena was isolated from other goats using fences and consisted of three pens. The subject goat was placed in the central pen, facing the other two pens. A stimulus goat, either a stablemate or a herd member, was placed in each of the other two pens. A speaker (Mackie Thump TH-12A) was located equidistant between the two stimulus goats and obscured by camouflage netting. A video camera (Canon LEGRIA) was mounted on a tripod above the speaker, orientated towards the subject, and a line was marked on the ground between the camera and the subject to facilitate the identification of the subject's gaze direction. The playback arena was located against a fence with vegetation behind the stimulus goats, to prevent other animals from moving behind the stimulus and minimize visual distractions to the subject. Playback presentations were controlled by an experimenter located approximately 10 m behind the subject and obscured from the subjects' view. Playback treatments consisted of two different calls from the same individual, separated by 10 s of silence, during which time the subject could look towards either of the stimulus goats.

### 2.4. Playback Series One

During Playback Series One, each subject was presented with a choice between its stablemate and a random herd member (the same goat throughout the series). Subject goats each received three treatments in the following order: (i) calls of the stablemate; (ii) calls of the herd member, while the side where the stablemate was presented (right or left pen, determined randomly) remained unchanged; and (iii) calls of the stablemate again, after exchanging the presentation sides of the stablemate and herd member. The first treatment was presented on one day and the remaining two were presented 6 days later, on the same day, a minimum of 2 h apart.

### 2.5. Playback Series Two

To determine if goats were capable of cross-modal recognition of familiar individuals, in general, or if it was restricted to closer social partners, a second series of playbacks was conducted. During the second series of playbacks, subjects were presented with a choice between two random herd members (different stimulus goats to those used during Playback Series One). Each subject received two treatments: (i) calls of one of the presented herd member, and (ii) calls of the other herd member, while the presentation sides remained unchanged. These two treatments were presented on the same day.

### 2.6. Playback procedure

The two stimulus goats were led from the field into the arena by experimenters and tethered in their enclosures on either side of the arena. The subject goat was then led into the central enclosure and tethered so that it faced the speaker and video camera, but was able to turn its head to the sides. Subject goats were allowed to habituate to the presentation arena for approximately 5 min before presentations. As far as possible, the subject was looking neutrally towards the camera when the first of the playback calls was given (at the beginning of the playback), and not directly at either stimulus goat. Presentations were filmed for later analysis. Stimulus goats did not vocalize during presentations and were not seen to show behaviours such as sudden movements that might attract the attention of the subject. Subjects were not rewarded during presentations. All goats were released back into the field with the herd at the end of the presentation.

### 2.7. Video analysis

Videos of the experiments were analysed by an observer who was blind to the presentation type. For each call played back (*n* = 2 per playback), the observer recorded two measures of responses: (i) the latencies from the onset of the call for the subject to look at each of the stimulus goats; and (ii) the duration of time spent looking at each goat, for 10 s from the onset of the call. This resulted, for each measure of response, in two values per call played back, one for each stimulus goat (i.e. four values for each playback). Looking at a stimulus goat was defined as orienting the head towards an individual in the area of binocular vision, approximately 60° along the midline of the head [42].

### 2.8. Statistical analysis

In order to investigate if subjects were able to attribute the calls played back to the congruent stimulus goat, we compared their latency to look and duration of time spent looking at the congruent and incongruent individuals using linear mixed effects models (LMM; lmer function, lme4 library) in Rv. 3.2.2 (R Development Core Team, 2015). The congruent individual was defined as the stimulus goat whose calls were played back and the incongruent individual was the other stimulus goat. Because the Playback Series One (stablemate versus herd member; *n* = 30 playbacks, 10 goats) and Two (two herd members; *n* = 20 playbacks, 10 goats) were carried out at different times and using different herd members as stimuli, their data were analysed separately. The latency to look and duration of time spent looking were fitted as dependent variables (two separate models for each playback series). Latency values in which the subject did not look at a given stimulus goat (*n* = 63 values for the first series and *n* = 37 values for the second series) were omitted from the analyses (e.g. if the subject did not look at the incongruent individual at all during a playback, no latency to look was included for this stimulus goat). This approach is more conservative than attributing a latency corresponding to a maximum possible value. In addition, latency values of 0 (*n* = 6 values for the first series and *n* = 6 values for the second series), indicating that the subject was already looking at the stimulus goat when the call started were omitted in order to control for initial side biases. In total, we thus included *n* = 51 latency values for the first series of playbacks and *n* = 37 latency values for the second series, whereas all duration values were included (*n* = 120 values: 3 playbacks × 2 calls × 2 stimulus goats × 10 subjects for the first series of playbacks; and *n* = 80 values: 2 playbacks × 2 calls × 2 stimulus goat × 10 subjects for the second series). The type of stimulus goat (congruent individual—corresponding to the playback; or incongruent—other individual) was included as a fixed effect, in order to compare the latency to look and duration of time spent looking at each of these goats. In the two models carried out on the first series of playbacks, the caller category (stablemate or herd member), as well as the interaction between caller category and type of stimulus goat (congruent or incongruent), were included as fixed factors, to test if the ability of subjects to attribute calls to the congruent stimulus goats differed between playbacks of stablemate and herd member calls. When it was not significant, the interaction term was removed from the models [43]. Two control factors were also included in all models: (i) because each playback consisted of two different calls from the same individual, we included the call number (1 or 2) as a fixed factor to control for any order effect; and (ii) the side (right or left) where the congruent individual was situated was included as a fixed factor to control for potential side biases. Finally, all models included as a random factor the playback number (1–5 for each goat) nested within the subject identity, in order to control for repeated measurements of the same subjects within and between playbacks, and for differences between playbacks (as four values per playbacks were included: 2 calls × 2 stimulus goats).

We checked the model residuals graphically for normal distribution and homoscedasticity. Models were fit with restricted maximum-likelihood method (RELM). The significance level was set at *α* = 0.05.

## 3. Results

**Figure 2.**
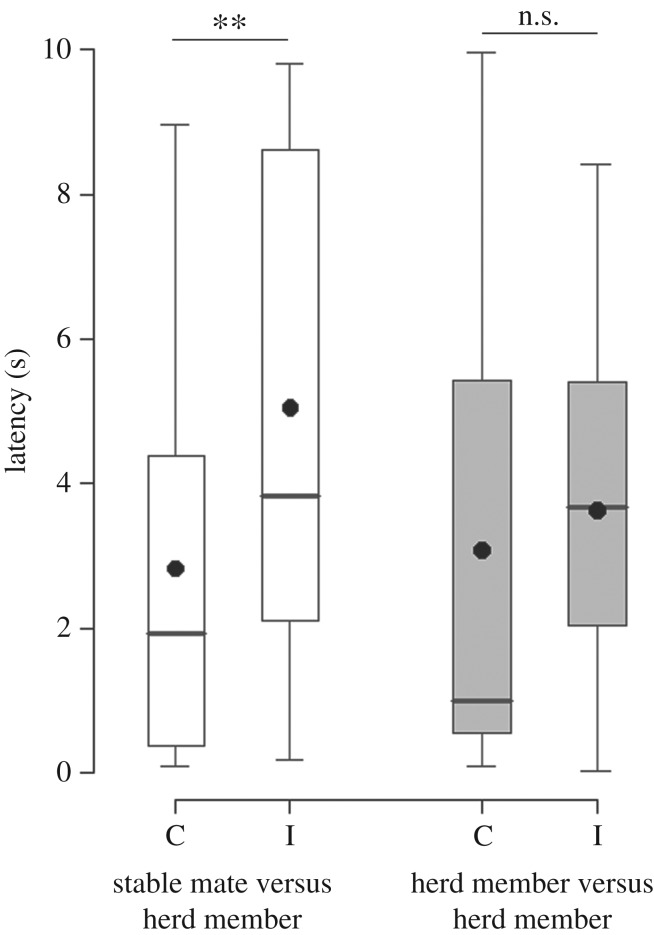
Latency to look. Latency to look at the congruent (C) or incongruent (I) stimulus goat during the first series of playbacks (stablemate versus herd member; in white) and during the second series of playbacks (herd member versus herd member; in grey), (box plot: the horizontal line shows the median, the box extends from the lower to the upper quartile and the whiskers to 1.5 times the interquartile range above the upper quartile or below the lower quartile; the black circles indicate the means; *n* = 10 goats; linear mixed effects models: **p < 0.01, n.s. , non-significant).

**Figure 3.**
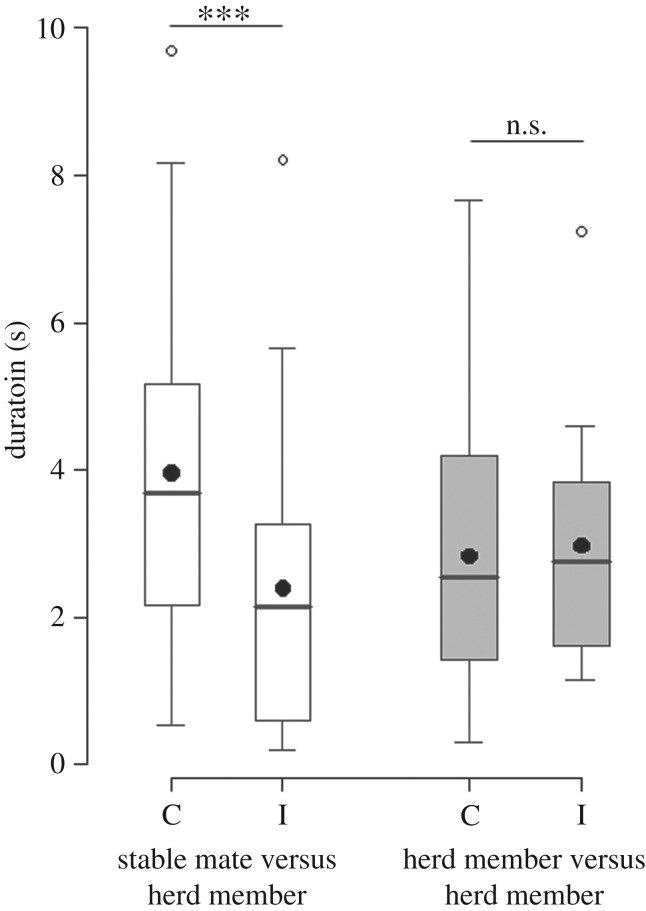
Duration of looks. Duration of time spent looking at the congruent (C) or incongruent (I) stimulus goat during the first series of playbacks (stablemate versus herd member; in white) and during the second series of playbacks (herd member versus herd member; in grey), (box plot: the horizontal line shows the median, the box extends from the lower to the upper quartile and the whiskers to 1.5 times the interquartile range above the upper quartile or below the lower quartile; empty circles indicate outliers; the black circles indicate the means; *n* = 10 goats; linear mixed effects models: ***p < 0.001, n.s., non-significant).

Subjects presented with a choice between their stablemate and a herd member (Playback Series One) looked faster (*Z* = 2.61, *n* = 51 latencies, *p* = 0.009; figure 2) and for a longer duration (LMM: *Z* = −3.37, *n* = 120 durations, *p* = 0.0008; figure 3) at the congruent compared to the incongruent stimulus goat, regardless of whether the calls played were those of the stablemate or herd member (i.e. the caller category and the interaction between caller category and type of stimulus goat had no effect on the results; caller category: latency, *Z* = 0.58, *p* = 0.56; duration, *Z* = −0.30, *p* = 0.76; interaction term: latency, *Z* = −1.21, *p* = 0.23; duration, *Z* = 1.51, *p* = 0.13). However, when presented with a choice between two random herd members (Playback Series Two), the subjects did not behave differently towards the congruent and incongruent stimulus goat (latency: *Z* = 0.68, *n* = 37 latencies, *p* = 0.50; figure 2; duration: *Z* = 0.14, *n* = 80 durations, *p* = 0.89; figure 3). To summarize, goats looked faster and for longer at the congruent compared to the incongruent stimulus goat, only when presented with a choice between their stablemate and another herd member (electronic supplementary material, Video S1). When presented with two random, less familiar, herd members, goats did not show a preference to look towards either individual.

## 4. Discussion

Using a preferential looking paradigm, we examined cross-modal correspondence between auditory and visual cues during individual recognition in goats. We hypothesized that, upon hearing a pre-recorded call, goats would look more rapidly and for longer towards the individual perceived as the source of the call. Subjects looked sooner and for longer towards the congruent stimulus goat when differentiating between a stablemate and a herd member. Therefore, goats are capable of cross-modal recognition of familiar social partners. However, subjects did not show a preference to look towards a particular individual when presented with two less familiar herd members. Further, goats appeared to exclude their stablemate as a potential caller when they heard the call of another individual. This suggests that goats may use inferential reasoning [44,45] during identification of conspecific callers. The circumstances in which cross-modal recognition occurs, and how individuals interpret signals has the potential to reveal how recognition systems evolve. The ways in which animals acquire information and perceive others provides a deeper understanding of animal cognition in general [9,10,44]. Our results show that familiarity influences cross-modal recognition with goats showing cross-modal recognition of close social partners but not of less familiar individuals, and that goats potentially use inference when processing conspecific signals.

We found that goats are capable of integrating information across two sensory modalities during recognition tasks, and are likely to possess internal templates or representations of other individuals comprising multimodal information. Further, while previous studies on goats have focused on vocal recognition in mother–offspring dyads [2], we found that adult goats are capable of cross-modally recognizing adult social partners. The recognition patterns that we observed are consistent with the social structure of these goats. In natural settings, goats typically forage in small groups during the day and congregate in larger groups overnight [27]. At the study site, the grouping pattern is somewhat reversed, i.e. goats forage in larger groups during the day and are stabled in smaller groups at night. However, it is somewhat surprising that goats did not appear to show cross-modal recognition of the two herd members because, although being less familiar than the stablemate, herd members had previous interactions with the subjects. In capuchin monkeys (*Cebus apella*) the ability to match pairs of images of conspecific faces also appears to be influenced by familiarity [46]. Capuchins performed better at matching images of familiar faces than unfamiliar faces, but were equally able to match faces of individuals living in their own social group and those living in a neighbouring group that they had daily visual and vocal access to. The ability of goats to cross-modally recognize stablemates, but not other herd members is potentially owing to very high familiarity and more frequent social interactions between stablemates than with other herd members.

It is often difficult to distinguish between individual recognition and class-level recognition, in which receivers learn individually distinctive characteristics of signallers and associate these with inferred class-specific information about them [10]. Proops *et al.* [9] proposed that, in order to demonstrate individual recognition, a paradigm must show that discrimination operates at an individual level and that there is a matching of current sensory information with stored information about that specific individual. Our results show that goats are capable of associating a stablemate's vocalization with visual information about this individual, which they have previously acquired. However, because goats did not show an association between calls and visual cues for other, less familiar herd members, it is difficult to determine if what we observed is class recognition (stablemate versus other) or individual recognition mediated by familiarity and the opportunity to learn other individuals' unique traits. In either case, goats appear to display cognitive representations of close social partners.

While vocal recognition of conspecifics has been demonstrated in goats [2], it is unlikely that the sound of the caller alone was sufficient to elicit the response observed. During presentations, the sound source was equidistant between individuals and they remained silent during presentations. Another modality of information, such as visual and/or olfactory, was required to provide the necessary cues for the subject to look towards the congruent individual. In our experimental design, the subject potentially had access to both visual and olfactory information about the stimulus goats. However, previous research that examined olfactory recognition in goats and sheep, particularly in mother–offspring recognition, found that close contact (i.e. less than 1 m) is necessary for the successful use of olfactory cues [47]. The subject and stimulus individuals in the current experiment were separated by at least 1.85 m, suggesting that subjects were more likely to be using visual–auditory cross-modal matching than olfactory–auditory.

One possible explanation for the association between a stablemate's call and looking towards that individual is a preference for looking towards familiar individuals. Domestic dogs fixate more often on the familiar faces of both conspecifics and humans than on unfamiliar faces [48]. Similarly, rhesus macaques fixate more rapidly on familiar conspecific faces than unfamiliar individuals [49]. In the first series of presentations, subjects did look towards the congruent individual faster and for longer than the incongruent individual when choosing between a stablemate and a herd member. However, subjects looked more at the congruent individual, regardless of whether they were the stablemate or not, indicating that goats were not simply looking towards the most familiar individual.

The current limited body of the literature on cross-modal recognition can provide insights into the factors that may lead to the evolution of cross-modal recognition [38]. These species all display extended social relationships with conspecifics [9,11,13,14,18], while some of those that have been shown to have cross-modal recognition of humans have a long history of domestication, i.e. horses [15,16] and dogs [17,50]. Goats appear to show similar traits to these species, with complex social relationships [27], as well as good physical and social cognitive abilities [20,22,25]. Examination of cross-modal recognition of humans by goats would be informative, as would investigation of a broader range of taxa, to determine if cross-modal recognition is associated with particular social or cognitive traits.

In the first series of presentations, when presented with a stablemate and a herd member, goats looked faster and for longer towards both the stablemate and the herd member after hearing their respective calls. However, in the second series of presentations, goats were unable to identify the caller when choosing between two herd members. This suggests that the choice to look towards the herd member in the first series of presentations may be based on an understanding that the call did not originate from the stablemate. This differential response to herd member calls between the two series suggests that goats may be capable of forming associations between auditory and visual cues through inferential reasoning, particularly ‘inference by exclusion’ [44]. Inference by exclusion involves the selection of the correct alternative by logically excluding other potential alternatives. Inference by exclusion has been proposed as a way by which animals may deal with inconsistent or incomplete information in their environments [44]. Previously, goats have been shown to use indirect information to locate food during an object-choice task, suggesting that goats potentially use inferential reasoning in other cognitive tasks [45]. In a social context, inference by exclusion is likely to allow individuals to acquire new associations, such as cross-modal linkages, without directly interacting with all individuals. Further experiments designed to explicitly examine inferential reasoning are needed to determine if goats possess this cognitive ability.

Using a preferential looking task, Proops & McComb [16] found that horses did not look towards unfamiliar humans when presented with their voice and a choice between the unfamiliar individual and a familiar handler. They suggest that this might indicate an inability to infer that an unknown voice originates from an unknown individual. Alternatively, they suggest that individuals might not be motivated to respond to a stranger. Further investigation into inference by exclusion in the recognition of other individuals, and its potential role in learning and the formation of cross-modal cognitive representations, would provide a greater understanding of how animals acquire information and perceive others.

Our results indicate that goats are likely to form cross-modal cognitive representations of conspecifics, and particularly of close social partners. However, the results of this study should be considered in light of its limited sample size. A larger sample size in Playback Series Two may also have shown that goats do cross-modally recognize less familiar individuals. Future studies of cross-modal recognition in goats should explore how individuals acquire information about conspecifics, including further exploring the extent to which familiarity influences the presence and accuracy of recognition abilities, as well as cross-modal recognition of other species such as humans. Further, while we attempted to control for side bias and to limit habituation, future studies could include additional suitable controls for these potential effects (e.g. [16]).

## 5. Conclusion

In conclusion, these results suggest that goats are capable of cross-modal recognition, and that the ability to recognize individuals is influenced by the level of familiarity between conspecifics. It is likely that, when recognizing close social partners, goats use cognitive templates that integrate information from multiple sensory modalities [9,10]. Further, goats appear to use inference by exclusion when processing social signals [44,45]. This may allow individuals to acquire new associations without requiring comprehensive investigation of other individuals. By examining cross-modal recognition in a diverse array of taxa, we can develop an understanding of the degree to which species can integrate information from multiple sensory modalities, and reveal insights into learning and the evolution of animal communication.

## Data accessibility

Data available from the Dryad Digital Repository: http://dx.doi.org/10.5061/dryad.gp8ck [52].
